# Prevention of unplanned endotracheal extubation in intensive care unit: An overview of systematic reviews

**DOI:** 10.1002/nop2.1317

**Published:** 2022-08-15

**Authors:** Jinhua Wu, Zhili Liu, Danqiao Shen, Zebing Luo, Zewei Xiao, Yeling Liu, Haixing Huang

**Affiliations:** ^1^ Shantou University Medical College Shantou China; ^2^ Shantou University Medical College Affiliated First Hospital Shantou China; ^3^ Cancer Hospital of Shantou University Medical College Shantou China

**Keywords:** intensive care unit, intratracheal, intubation, nursing care, systematic review, unplanned extubation

## Abstract

**Aims:**

This study was performed to identify and summarize systematic reviews focusing on the prevention of unplanned endotracheal extubation in the intensive care unit.

**Design:**

Overview of systematic reviews.

**Methods:**

This overview was conducted according to the Preferred Reporting Items for Overviews of Systematic Reviews, including the harms checklist. A literature search of PubMed, the Cochrane Library, CINAH, Embase, Web of Science, SINOMED and PROSPERO was performed from January 1, 2005–June 1, 2021. A systematic review focusing on unplanned extubation was included, resulting in an evidence summary.

**Results:**

Thirteen systematic reviews were included. A summary of evidence on unplanned endotracheal extubation was developed, and the main contents were risk factors, preventive measures and prognosis. The most important nursing measures were restraint, fixation of the tracheal tube, continuous quality improvement, psychological care and use of a root cause analysis for the occurrence of unplanned endotracheal extubation.

**Conclusions:**

This overview re‐evaluated risk factors and preventive measures for unplanned endotracheal extubation in the intensive care unit, resulting in a summary of evidence for preventing unplanned endotracheal extubation and providing direction for future research.

**Trial registration details:**

The study was registered on the PROSPERO website.

## 1 INTRODUCTION

Unplanned endotracheal extubation (UEE) is defined as any dislodging of an endotracheal tube from the trachea that is not intentional or ordered by a healthcare professional (Klugman et al., 2020). UEE can lead to serious complications such as asphyxia, malignant arrhythmia and aspiration pneumonia (Chao, Sung, et al., 2017) and serves as an important and sensitive indicator of quality of care (Chuang et al., 2015). A meta‐analysis of 34 studies shows that the pooled prevalence of UEE is 6.69% (Li et al., 2022). Notably, 70% of UEE events are preventable (da Silva & Fonseca, 2020).

## 2 BACKGROUND

Care bundles are considered as valuable and proven to affect care quality (da Silva et al., 2022), but finding enough systematic and valid evidence to achieve a high bundle fidelity is challenging. In recent years, growing numbers of original studies and systematic reviews are focusing on UEE; however, the quality of available nursing studies related to UEE prophylaxis is mixed, and single studies or systematic reviews provide only scattered evidence on individual issues with no currently available relevant clinical practice guidelines or accepted best practices (Crezeé et al., 2017). A systematic review synthesizes evidence at a higher level and contains a richer and more comprehensive body of information with greater clinical utility (Ji et al., 2020). In the current situation of the growing number of systematic reviews related to UEE, an overview of systematic reviews is recommended as a suitable and logical step for comparison and contrast of individual reviews (Connolly et al., 2015) and can improve the scientific accuracy and validity of evidence‐based practice for preventing UEE (Li et al., 2019). This overview of systematic reviews related to prevention of UEE was conducted according to the Preferred Reporting Items for Overviews of Systematic Reviews, including the harms (PRIO‐harms) checklist (Bougioukas et al., 2018).

Research questions:

- What are the current systematic reviews on UEE in the intensive care unit?
- What are the best measures to prevent UEE in the intensive care unit?

## 3 METHODS

The protocol of this overview has been registered on the PROSPERO website, and the registration number is CRD42021253097. This overview of systematic review was reported according to the PRIO‐harms.

### 3.1 Literature inclusion and exclusion criteria

#### 3.1.1 Literature inclusion criteria

- The study subjects were intensive care unit patients.
- The study topics were related to unplanned extubation of tracheal intubation in the intensive care unit, including risk factors and preventive measures for unplanned extubation of tracheal intubation, etc.
- The type of literature was systematic review or meta‐analysis of all study designs.
- Both English and non‐English language articles were considered.

#### 3.1.2 Exclusion criteria

- Systematic review proposal.
- Literature for which the full text was not available.

### 3.2 Search strategies

#### 3.2.1 Database

Computer searches the following databases: PubMed, Cochrane Library, CINAHL, Embase, Web of science, SINOMED and PROSPERO.

#### 3.2.2 Search strategy

(“Airway Extubation”[MeSH Terms] OR “unplanned extubation*”[Title/Abstract] OR “accidental extubation*”[Title/Abstract] OR “spontaneous extubation*”[Title/Abstract] OR “self extubation*”[Title/Abstract] OR “unexpected extubation*”[Title/Abstract] OR “inadvertent extubation*”[Title/Abstract] OR “unintentional extubation*”[Title/Abstract] OR “UE”[Title/Abstract]) AND (“Intensive Care Units”[MeSH Terms] OR “Critical Care”[MeSH Terms] OR ‘critical illness*’[Title/Abstract] OR ‘critical ill patient*’[Title/Abstract] OR ‘Intensive Unit*’[Title/Abstract] OR ‘critically ill’[Title/Abstract]) AND (‘Review’[Publication Type] OR ‘systematic review’[Publication Type] OR ‘Meta‐Analysis’[Publication Type]) AND (‘January 1, 2005’[PDAT]:”June 1, 2021”[PDAT]).

### 3.3 Search methods

The search was conducted in June–July 2021 by the first author and the second author, and we searched the literature from the time the database was built to June 1, 2021, using a subject term plus free word search combined with a literature traceability method. A pre‐search was conducted in the PubMed database prior to the formal search, and the research team discussed and analysed the search results and revised the search strategy before conducting a full formal search.

### 3.4 Selection of reviews

Two researchers independently selected the studies identified by the search strategies. The researchers eliminated a study based on its title and abstract and then independently assessed the eligibility of the remaining studies based on their full text. Disagreement was resolved by consensus.

### 3.5 Data extraction

Two researchers trained in Evidence‐Based Nursing independently screened the literature, imported their respective searches into EndNote X9 software (Clarivate, London, UK) for initial screening to exclude duplicates, manually ranked the literature to remove duplicates and independently extracted information based on the inclusion criteria. The extracted data were then compared, and a third party was asked to arbitrate when disagreement was encountered to ensure the accuracy of the information. In the case of non‐English articles, two native speakers translated them independently and the translation articles were verified by a third party. Information was extracted by author, year, country/region, institution, content/subject, type of study included, number of studies included, quality assessment tool and primary outcome by creating a uniform form. The form was validated on a trial basis before the reviewers began to independently collect data. Two reviewers extracted data from both articles, compared their answers and discussed their findings to ensure that they both interpreted the questions in the same way.

### 3.6 Quality assessments of included reviews

Systematic reviews considered eligible for inclusion were appraised for their methodological quality using the Joanna Briggs Institute (JBI) Critical Appraisal Checklist for Systematic Reviews and Research Synthesis (Aromataris et al., 2015). The JBI Critical Appraisal Checklist is a tool that evaluates both quantitative and qualitative reviews based on principles common across accepted quality assessment tools. It contains 11 questions related to the systematic review method, and each question is answered as “yes,” “no,” “unclear” or “not applicable.” Two reviewers worked in pairs again and finished the assessment independently in duplicate.

Before beginning an independent assessment, the two reviewers piloted the form, testing it on two studies; these reviewers then compared their results and discussed the acceptable level of information required to decide whether a review fits the criteria and what is considered “unclear.” The systematic review was considered of insufficient quality for inclusion and thus excluded at this step if at least one of the following was rated “unclear” or “not applicable”: “clear review questions,” “appropriate inclusion criteria,” “appropriate search strategy” or “appropriate critical appraisal criteria.”

### 3.7 Data analysis and synthesis

The included literature was read and analysed iteratively by two researchers, and the extracted data were presented in tabular form to consolidate and descriptively analyse the findings and form the results. Because of the heterogeneity of the included systematic reviews/meta‐analyses and the fact that data of results from some studies were not merged, only a descriptive analysis was performed. In addition, evidence related to prophylaxis for UEE was graded for quality using the JBI Levels of Evidence and Grades of Recommendation (Munn et al., 2014). The recommended strengths were determined through group discussion based on the “feasibility, suitability, significance and effectiveness” of the evidence.

### 3.8 Ethical Statement

The Research Ethics Committee approval was not required.

## 4 RESULTS

### 4.1 Search results

**FIGURE 1:**
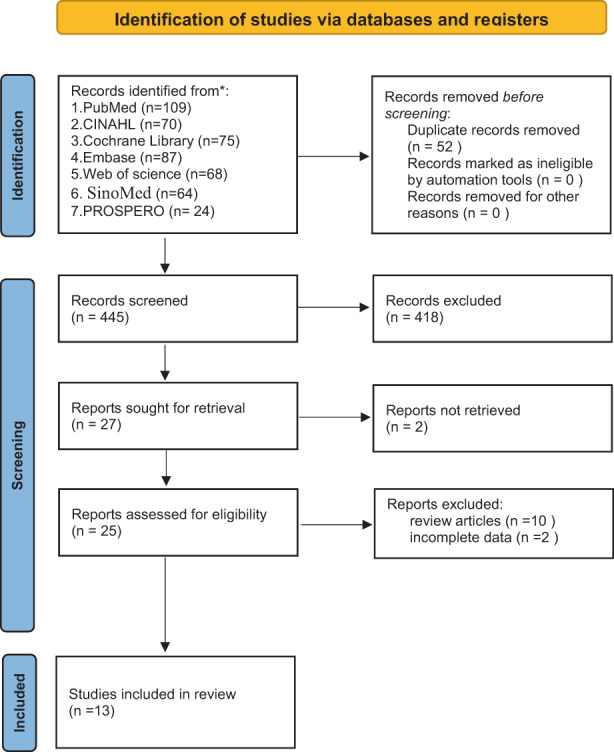
Preferred Reporting Items for Systematic Reviews and Meta‐Analyses (PRISMA) flowchart 2020 describing the study selection process

The screening process was reported using the Preferred Reporting Items for Systematic Reviews and Meta‐Analyses 2020 (Pas et al., 2021) flow chart (see Figure 1). In total, 497 articles were identified in the initial search (PubMed, *N* = 109; CINAH, *N* = 70; Cochrane Library, *N* = 75; Embase, *N* = 87; Web of Science, *N* = 68; SINOMED, *N* = 64; and PROSPERO, *N* = 24). We screened the titles and abstracts and retrieved 25 full‐text articles. After full‐text review, a further 12 articles did not meet the eligibility criteria. Finally, 13 studies (Ai et al., 2018; da Silva et al., 2017; Gardner et al., 2005; Ge, Xu, et al., 2014; Ge, Zhu, et al., 2014; Kiekkas et al., 2013; Lai et al., 2014; Liu et al., 2019; Lucas da Silva & de Carvalho, 2010; Silva et al., 2013; Yan et al., 2016; Zhu et al., 2014) were included in this overview.

### 4.2 Characteristics of included studies

Meta‐analyses were performed on 6 of the 13 included systematic reviews (Ai et al., 2018; da Silva et al., 2017; Gardner et al., 2005; Liu et al., 2019; Yan et al., 2016; Zhu et al., 2014); the others underwent descriptive analyses. The methodological aspects of systematic review production mainly involved the following four methodological quality assessment tools: The Newcastle–Ottawa Scale, the Cochrane Collaboration Risk of Bias Tool, the Australian JBI literature quality assessment criteria and the Jadad scale. In total, 217 original studies were included in this study, the research themes of which were mainly related to risk factors, preventive measures and outcome indicators for UEE. The study population was divided into adults, children, young children and infants. Nine studies involved adults, and two studies each involved neonates and children. The original studies included in the literature were all from 2018 and earlier. Four articles were from the same author (da Silva & Fonseca, 2012; da Silva et al., 2017; Lucas da Silva & de Carvalho, 2010; Silva et al., 2013). The original studies included in the systematic review were mainly cohort studies, case–control studies and randomized controlled studies, with no mention of qualitative studies involving patient experience as detailed in Table 1, which shows the characteristics of the included systematic reviews.

**TABLE 1:** Characteristics of included systematic reviews

Systematic reviews	Years searched	Subjects	Primary studies	Study population	Synthesis method	Quality assessment tools	Main conclusions
Yan et al. (2016)	Jan 1998–Jan 2014	Psychological care on UE	8 RCT	Adults	Meta‐analysis	Jadad	Psychological care can reduce the incidence of UE
Liu et al. (2019)	Database built to Oct 2018	Root cause analysis on UE	12 Cohorts	Adults	Meta‐analysis	NOS	Root cause analysis can reduce the rate of UE
Ge, Xu, et al. (2014)	Database built to Apr 2013	Risk factors	26 Cohorts	Adult	Narrative synthesis	JBI Evaluation Tool	Physical restraint in patients with a GCS > 9 increases the risk of UE by a further 6.16 times. and other risk factors for UE are: Male, delirium, transoral intubation, midazolam sedation
Ge, Zhu, et al. (2014)	Database built to Apr 2013	Risk factors	23 (RCT/Cohorts)	Adult	Narrative synthesis	JBI evaluation tool	The high‐risk occurrence times for UE are night, 1 h before and after shift changeover and ventilator withdrawal stage.
Zhu et al. (2014)	Database built to Jun 2012	Prevention of UE	8 RCT, cohorts, CCT	Adults	Meta‐analysis	CCRBT/NOS	Restraint on its own is not effective in preventing UE, and relevant nursing interventions in conjunction with patient restraint are more effective than restraint alone
Ai et al. (2018)	Database built to Sep 2017	Risk factors	10 Cohorts, CCT	Adults	Meta‐analysis	NOS	The patient was confused, male, physically limited, with a high GCS and an APACHE II score is a statistically significant risk factor for unplanned extubation in adult patients in ICU
da Silva and Fonseca (2012)	Jan 1950–May 2011	Risk factors and prevention of UE	50 Queue, CCT	Adult	Narrative synthesis	NOS	Standardized endotracheal tube fixation procedures and continuous quality improvement; development of a decannulation programme for early identification of extubation pointers to maintain a certain ratio of nurses and training of nurses can reduce UE
Kiekkas et al. (2013)	Jan 1990–Apr 2012	Risk factors and prevention of UE	34 Cohorts, CCT.	Adult	Narrative synthesis	None	Risk factors are agitation, monitoring, nurse–patient ratio and catheter fixation, physical restraint remains controversial, and reintubation is an important outcome indicator
Gardner et al. (2005)	1993–2003	Tracheal intubation fixation	7 RCT, cohorts, CCT.	Adults	Meta‐analysis	None	There is no optimal method of ET fixation and more RCTs related to ET need to be conducted and costs and time to care evaluated
Silva et al. (2013)	Jan 1950–Jan 2012	Risk factors and prevention of UE	15 Cohorts, CCT	Newborn	Narrative synthesis	NOS	UE rates ranged from 0.14–5.3/100 intubation days, with risk factors such as irritability, poor fixation of the tracheal tube and bedside handling. Fixation of the catheter is controversial and reintubation rates range from 8.3%–100%
Lai et al. (2014)	Database built to Sep 2014	Tracheal intubation fixation	5 RCT	Newborn	Narrative synthesis	CCRBT	No studies reported the incidence of reintubation or tracheal tube dislocation, other outcome indicators were: mortality, incidence of perioral skin trauma, no optimal fixation method for neonatal ET
Lucas da Silva and de Carvalho (2010)	Jan 1966–Mar 2009	Risk Factors and Prevention of UE	11 Cohorts, CCT	Children	Narrative synthesis	None	The reintubation rate for unplanned extubation in children is 22%–78%. UE measures include mainly restraint, sedation, fixation of the tracheal tube and continuous quality improvement
da Silva et al. (2017)	Jan 2010–Jun 2016	Risk factors	8 Cohorts, CCT	Children	Meta‐analysis	NOS	Risk factors: Bedside operations, patient agitation and endotracheal intubation care, optimal threshold for UE unknown

Abbreviations: CCRBT, Cochrane Collaboration's Risk of Bias Tool； CCT, case–control trial； ET, endotracheal tube; ICU, intensive care unit; JBI, Joanna Brigg's Institute; NOS, Newcastle‐Ottawa Scale; RCT, randomized controlled trial； UE, unplanned extubation.

### 4.3 Methodological quality of the included systematic reviews

Methodological quality was determined using a critical evaluation checklist tool by the JBI (see Table 2, which shows the results of the critical appraisal as a whole). The critical appraisal of the methodological quality of the included reviews varied across the studies, with three studies meeting 100% of the criteria, six studies meeting >90% of the criteria and four studies meeting >81% of the criteria. The most commonly inapplicable item was related to the assessment of publication bias (Q9), which was assessed in 7 (54%) of the 13 studies. The most commonly unmet item was recommendations for policy and/or practice supported by the reported data (Q10), which was assessed in 6 (46%) of the 13 studies. Other items (Q1–Q8 and Q11) were fulfilled in all or nearly all the studies.

**TABLE 2:** The methodological quality of systematic reviews assessed by the JBI critical appraisal checklist for systematic reviews and research syntheses

Systematic reviews	Q1	Q2	Q3	Q4	Q5	Q6	Q7	Q8	Q9	Q10	Q11
Yan et al. (2016)	Yes	Yes	Yes	Yes	Yes	Yes	Yes	Yes	Yes	Yes	Yes
Liu et al. (2019)	Yes	Yes	Yes	Yes	Yes	Yes	Yes	Yes	Yes	No	No
Ge, Xu, et al. (2014)	Yes	Yes	Yes	Yes	Yes	Yes	Yes	Yes	NA	No	Yes
Ge, Zhu, et al. (2014)	Yes	Yes	Yes	Yes	Yes	Yes	Yes	Yes	NA	No	Yes
Zhu et al. (2014)	Yes	Yes	Yes	Yes	Yes	Yes	Yes	Yes	Yes	No	Yes
Ai et al. (2018)	Yes	Yes	Yes	Yes	Yes	Yes	Yes	Yes	Yes	Yes	Yes
da Silva et al. (2012)	Yes	Yes	Yes	Yes	Yes	Yes	Yes	Yes	NA	Yes	Yes
Kiekkas et al. (2013)	Yes	Yes	Yes	Yes	Yes	Yes	Yes	Yes	NA	Yes	Yes
Gardner et al. (2005)	Yes	Yes	Yes	Yes	Yes	Yes	Yes	Yes	Yes	No	Yes
da Silva et al. (2012)	Yes	Yes	Yes	Yes	Yes	Yes	Yes	Yes	NA	No	Yes
Lai et al. (2014)	Yes	Yes	Yes	Yes	Yes	Yes	Yes	Yes	NA	Yes	Yes
Lucas da Silva and de Carvalho (2010)	Yes	Yes	Yes	Yes	Yes	Yes	Yes	Yes	NA	Yes	Yes
da Silva et al. (2017)	Yes	Yes	Yes	Yes	Yes	Yes	Yes	Yes	Yes	Yes	Yes

Note: Questions: Q1. Is the review question clearly and explicitly stated? Q2. Were the inclusion criteria appropriate for the review question? Q3. Was the search strategy appropriate? Q4. Were the sources and resources used to search for studies adequate? Q5. Were the criteria for appraising studies appropriate? Q6. Was critical appraisal conducted by two or more reviewers independently? Q7. Were there methods to minimize errors in data extraction? Q8. Were the methods used to combine studies appropriate? Q9. Was the likelihood of publication bias assessed? Q10. Were recommendations for policy and/or practice supported by the reported data? Q11. Were the specific directives for new research appropriate?

Abbreviation: NA, Not applicable.

### 4.4 Results of data analysis

#### 4.4.1 Risk factors for UEE

Eight studies involved risk factors for UEE (Ai et al., 2018; da Silva et al., 2017; da Silva & Fonseca, 2012; Ge, Xu, et al., 2014; Ge, Zhu, et al., 2014; Kiekkas et al., 2013; Lucas da Silva & de Carvalho, 2010; Silva et al., 2013), which were male sex (Ai et al., 2018; Ge, Xu, et al., 2014), delirium (Ai et al., 2018; Ge, Xu, et al., 2014), restraint (Ai et al., 2018; Kiekkas et al., 2013), a higher Glasgow Coma Scale (GCS) score (Ai et al., 2018; Ge, Xu, et al., 2014), patient agitation (da Silva et al., 2017; Kiekkas et al., 2013), monitoring, the nurse–patient ratio and catheter fixation (Kiekkas et al., 2013; Silva et al., 2013), irritability (Silva et al., 2013), poor fixation (Silva et al., 2013), bedside handling (da Silva et al., 2017; Silva et al., 2013), endotracheal extubation manipulation (da Silva et al., 2017), APACHE II score (Ai et al., 2018) and times of high risk for UEE occurrence (at night, 1 h before and after shift changeover, and during ventilator withdrawal stage) (Ge, Zhu, et al., 2014).

#### 4.4.2 Preventive measures

Nine studies (da Silva & Fonseca, 2012; Gardner et al., 2005; Kiekkas et al., 2013; Lai et al., 2014; Liu et al., 2019; Lucas da Silva & de Carvalho, 2010; Silva et al., 2013; Yan et al., 2016; Zhu et al., 2014) focused on preventive measures to reduce UEE. These measures mainly included psychological care, root cause analysis methods, restraint, securing the tracheal tube with suitable fixation methods, continuous quality improvement and management of sedation and risk factors.

- Psychological care and root cause analysis.

Psychological care and root cause analysis can reduce the incidence of UEE. A meta‐analysis (Yan et al., 2016) that included eight randomized controlled trials showed that psychological care reduced the incidence of UEE. Another meta‐analysis (Liu et al., 2019) that included 12 cohort studies showed that root cause analysis reduced the incidence of UEE.

- Restraint.

The use of restraints to prevent UEE has been controversial. Restraint in patients with a GCS score of >9 increased the risk of UEE by a further 6.16 times (Ge, Xu, et al., 2014). Restraint on its own was not effective in preventing UEE, and relevant nursing interventions in conjunction with restraint were more effective than restraint alone (Zhu et al., 2014). Restraint was one effective way to reduce UEE (Kiekkas et al., 2013).

- Securing the tracheal tube with suitable fixation methods.

An optimal method of tracheal intubation fixation has not yet been established. Two studies included tracheal intubation fixation; one was for adults published in 2005 (Gardner et al., 2005) and the other for newborns in 2014 (Lai et al., 2014). Many methods for fixation of tracheal intubation are both local and specific, and there is not enough information to prove which is the best method for tracheal intubation fixation for adults. And more randomized controlled trials related to tracheal intubation fixation need to be carried out to evaluate the cost and duration of treatment (Gardner et al., 2005). There is a lack of evidence to determine the most effective and safe method of stabilizing the tracheal tube in ventilated newborns (Lai et al., 2014).

- Continuous quality improvement.

Continuous quality improvement has been proved an effective method to reduce the UEE. Two studies show that continuous quality improvement can reduce the incidence of UEE not only in adults but also in children (da Silva & Fonseca, 2012; Lucas da Silva & de Carvalho, 2010).

- Management of sedation and risk factors.

Sedation management and enhanced monitoring of high‐risk groups and high‐risk time periods can reduce the incidence of UEE (Ge, Xu, et al., 2014). One study revealed the need to enhance monitoring during high‐risk times to prevent UEE (Ge, Zhu, et al., 2014). Although many studies have focused on UEE, few have examined UEE prevention strategies and related clinical trials (da Silva & Fonseca, 2012). In the present overview, there was a lack of UEE risk assessment tools and studies related to the timing and frequency of assessment among the included studies.

#### 4.4.3 Summary of evidence and recommended levels

Based on the JBI Levels of Evidence and Grades of Recommendation, we compiled a summary of evidence about the results and levels of evidence of the studies in this overview (see Table 3). The evidence was graded into five levels based on the JBI evidence levels and recommendation levels. The strengths of evidence decrease from level 1–level 5, with level 1 indicating the highest evidence studies and level 5 being the lowest level of evidence. Based on the JBI FAME structure, the strength of recommendations was independently rated by two researchers who had systematically studied evidenced‐based nursing according to the validity, feasibility, appropriateness and clinical significance of the evidence, and they performed this rating in accordance with the JBI recommendation strength grading principle of the evidence. In cases of disagreement, a third person arbitrated the dispute. “A” represents a strong recommendation, and “B” represents a weak recommendation. The summary of evidence contains 20 pieces of evidence on risk factors, preventive measures and outcome indicators for UEE. Of these 20 pieces of evidence, 17 are Level 3, and 3 are Level 1, and the quality of the evidence was all moderate or high quality. The level of recommendation of the evidence was Grade A (strong recommendation) for 16 and Grade B (weak recommendation) for 4.

**TABLE 3:** Summary of evidence and recommended levels

Dimensionality	Item	Contents of the evidence	Evidence level	Recommended strengths
Risk factors	1	Bedside operations and endotracheal intubation care (Da Silva., 2017)	Level1	A
	2	Patient agitation (da Silva et al., 2017) and irritability (Silva et al., 2013)	Level3	A
	3	Poor fixation of the tracheal tube (Silva et al., 2013)	Level3	A
	4	The high‐risk occurrence times for UE are night, 1 h before and after shift changeover and ventilator withdrawal stage (Zhu et al., 2014).	Level3	A
	5	The patient was confused, male, physically limited, and with a high GCS (Ai et al., 2018)	Level3	A
	6	Monitoring, nurse–patient ratio and catheter fixation, physical restraint remains controversial (Kiekkas et al., 2013)	Level3	A
Preventions	1	Using the root cause analysis for unplanned extubation patients (Liu et al., 2019)	Level3	A
	2	Enhancing psychological care for endotracheal intubation patients (Yan et al., 2016)	Level3	B
	3	Regulating restraint and sedation management, increasing monitoring of high‐risk groups and high‐risk periods (Ge & Xu et al., 2014)	Level3	A
	4	To enhance restraint care and reduce adverse effects of restraint on patients and families, restraint should be used with caution and increased precautions for GCS scores ≥ 9 (Zhu et al., 2014)	Level3	A
	5	Standardization of endotracheal tube fixation procedures (da Silva et al., 2012).	Level3	B
	6	Develop an offline programme for early identification of extubation pointers (da Silva et al., 2012)	Level3	B
	7	Maintaining a certain ratio of nurses (Nurse‐to‐patient ratio of 1:1) (da Silva et al.^2012^; Lucas da Silva & de Carvalho, 2010)	Level3	B
	8	No optimal fixation method for neonatal ETT (Lai et al., 2014)	Level1	A
	9	Training Nurses (da Silva et al., 2012)	Level3	A
	10	Developing appropriate data tracking and data collection tools for unplanned extubation of tracheal tubes (Lucas da Silva & de Carvalho, 2010)	Level3	A
	11	Setting standards of procedures such as tracheal tube fixation, tube suctioning, patient hygiene, and transport (Lucas da Silva & de Carvalho, 2010)	Level3	A
	12	Carrying out continuous quality improvement (Lucas da Silva & de Carvalho, 2010)	Level3	A
Outcome indicator	1	Reintubation is an important outcome indicator (Kiekkas et al., 2013)	Level3	A
	2	Outcome indicators include: mortality, incidence of perioral skin trauma (Lai et al., 2014)	Level1	A

Note: The methods of grading and recommendations of the evidence: Evidence was graded for quality using the JBI Levels of Evidence and Grades of Recommendation (Munn et al., 2014). The recommended strengths were determined through group discussion based on the “feasibility, suitability, significance and effectiveness” of the evidence. Evidence level: The evidence level is divided into five levels: level 1 indicates the highest evidence studies, level 3 moderate evidence studies, and level 5 the lowest evidence studies. Recommended strengths: A represents the strong recommendation, and B represents the weak recommendation.

## 5 DISCUSSION

To our knowledge, this is the first overview of systematic reviews for UEE in the intensive care unit. We conducted a descriptive analysis of 13 pieces of literature related to UEE. The systematic review related to UEE was mainly concerned with two aspects: risk factors and preventive measures. After quality evaluation of the literature and evidence, 20 relevant pieces of evidence were compiled into evidence summaries. This evidence can provide a useful reference for clinical care and clinical nursing practice.

### 5.1 Risk factors

With regard to risk factors for UEE, there was general agreement that male sex, delirium, higher GCS scores (Ai et al., 2018; Ge, Xu, et al., 2014), restraint (Ai et al., 2018; Kiekkas et al., 2013), patient agitation (da Silva et al., 2017; Kiekkas et al., 2013), monitoring, the nurse–patient ratio and catheter fixation (Kiekkas et al., 2013; Silva et al., 2013), and bedside handling (da Silva et al., 2017; Silva et al., 2013) were important risk factors. However, the effects of sex and restraint on the tracheal tube have long been controversial. One study has suggested that the impacts of age and sex on tracheal intubation are unclear (Zhang & Liu, 2021), whereas the other has shown that UEE is more common in younger people (Kwon & Choi, 2017). In this study, two systematic reviews (Ai et al., 2018; Ge, Xu, et al., 2014) show that men are an important factor, probably because men are often physically stronger than women, and the tracheal tube can be more easily removed from young patients than from elderly patients and children because the young usually have more strength than the old and children. Whether restraint can prevent UEE is debated worldwide. Although restraint is still the preferred option (Zhang & Liu, 2021), UEE still occurs when restraint is used. The existence of this controversy may be due to inappropriate restraint; thus, the method of restraint and the pointer to constraint should be strictly controlled.

### 5.2 Prevention

#### 5.2.1 Further research is needed on the specific implementation of psychological care to prevent UEE

As modern medicine has changed from a “disease‐centred biomedical model” to a “human‐centred biopsychosocial medical model” (Miao et al., 2020), psychological care has gradually been emphasized (Chambers et al., 2018; Thomas et al., 2018) and has become an essential part of daily care. Psychological care can reduce the incidence of UEE and is often included in our nursing routines as a necessary care measure (Hay et al., 2019), and our study also confirms this. Psychological care is particularly important because of the unique environment of the intensive care unit and the difficulties in communicating with intubated patients. One study suggested that the patient's needs should be understood and met in a timely manner to relieve the adverse stimulation **(**Wang et al., 2019 **).** However, there is a lack of specific and compelling care measures on how to do psychological care. Further research is needed on the specific implementation of psychological care for patients with whom communication is difficult because of tracheal intubation.

#### 5.2.2 Methods and pointers for restraints should be strictly mastered

The effect of restraints on UEE has been considered controversial (Li et al., 2017; Luk et al., 2014). Despite that, clinical staff has included restraints as a preventive measure against UEE because they give medical staff a sense of security. The use of restraints in the ICU is very common throughout the world **(**Perez et al., 2019 **)**. In our study, Zhu et al., 2014 performed a meta‐analysis of four studies and found that restraint alone was not significantly effective in preventing UEE but that restraint combined with other nursing measures was superior to restraint alone (Level 3, Grade A recommendation). This may reveal one of the reasons for the uncertainty of the effect of restraint on UEE; the effect of restraint on UEE is in fact not absolute and should be determined on a case‐by‐case basis. So, we should master proper methods and pointers strictly for restraints to prevent UEE.

#### 5.2.3 Securing the tracheal tube with suitable fixation methods

Studies have shown that proper securing of the tracheal tube is an effective measure to prevent UEE (Silva et al., 2013; Wang et al., 2019). In recent years, however, few high‐quality studies have focused on fixation of the tracheal tube. Systematic reviews of fixation methods for tracheal intubation in adults and infants were conducted by Gardner et al. (Gardner et al., 2005) and Lai et al. (Lai et al., 2014) in 2005 and 2015, respectively, but these studies failed to identify the optimal fixation method for tracheal intubation because of the low quality and heterogeneity of the included literature. With advances in technology, catheter materials and fixation methods have long been updated and new fixation methods have emerged (Walters et al., 2018). Maybe, the optimal technique for fixation of the tracheal tube needs to be further explored. However, the fixation of tracheal intubation is the most fundamental measure, and there are advantages and disadvantages to various fixation methods. There is no best, but the most suitable fixation method should be taken according to the actual situation of the patient. In addition, there is a need for high‐quality original research on the latest fixation methods, and it would also be informative to pool the best evidence for tracheal intubation fixation in conjunction with the latest research.

#### 5.2.4 Insist on continuous quality improvement

In our study, carrying out continuous quality improvement (Level 3, Grade A recommendation) is a vital and effective measure in the prevention of UEE, resulting in a 24.1% absolute reduction rate in UEE and a 36.6% absolute reduction rate in associated cardiovascular failure (Klugman et al., 2020). A similar study shows that after 15 years of continuous quality improvements, the unplanned extubation rate decreased from 9.0/1000 ventilator days–1.36/1000 ventilator days (Chao, Lai, et al., 2017).

Summary of the evidence

Studies related to UEE are numerous and fragmented, but there are few such forms of evidence aggregation for systematic review. In our research, the evidence about UEE was summarized and marked with the level of evidence and recommendation. Specific evidence was extracted from three aspects of UEE: risk factors, prevention and outcome indicators, and the evidence extracted was all moderate (Level 3) or above, providing medical and nursing staff with a clear and definite basis of information for clinical application, and indicating methods for the prevention of UEE, which is of great clinical significance.

### 5.3 Implications for research

Enhancing psychological care for endotracheally intubated patients (Yan et al., 2016) (Level 3, Grade B recommendation) is an important measure to prevent UEE. However, previous studies related to psychological care lack specificity and are not clinically actionable, resulting in a Grade B recommendation. Further specific psychological care‐related prevention measures should be developed to facilitate clinical implementation. In addition, although identification of the risk of UEE is particularly important, no studies in this overview addressed UEE assessment tools or the frequency of assessment frequency aspect. A risk assessment tool should be developed at a later stage for the graded prevention of tracheal intubation in the intensive care unit.

### 5.4 Limitations

This overview of systematic reviews had two main limitations. First, we retrieved data from reviews instead of primary studies. The reviews could have had several weaknesses in methodological quality, which would have affected the findings of this overview. Thus, we assessed the methodological quality of the included reviews to identify weaknesses. When interpreting the evidence, the methodological quality was considered. Second, because of the heterogeneity of the literature included in the study, the data were not combined and the effect values could not be analysed.

## 6 CONCLUSIONS

This systematic review reassessed the risk factors and preventive measures for preventing UEE in the intensive care unit and developed an evidence summary with 20 pieces of evidence and recommendations, thus serving as a reference and providing evidence to support the construction of clinical practice guidelines. Moreover, it is recommended that future studies related to specific psychological care for patients with tracheal intubation, continuous quality improvement, different fixation methods for specific conditions of patients and strictly mastering restraint guidelines and methods can be carried out to prevent the occurrence of UEE.

## Data Availability

Data sharing is not applicable to this article as no new data were created or analyzed in this study.
